# Real-world experience of sorafenib maintenance after allogeneic hematopoietic stem cell transplantation for FLT3-ITD AML reveals high rates of toxicity-related treatment interruption

**DOI:** 10.3389/fonc.2023.1095870

**Published:** 2023-03-15

**Authors:** Sarah Morin, Federica Giannotti, Anne-Claire Mamez, Amandine Pradier, Stavroula Masouridi-Levrat, Federico Simonetta, Yves Chalandon

**Affiliations:** ^1^ Division of Hematology, Department of Oncology, Geneva University Hospitals, Geneva, Switzerland; ^2^ Translational Research Center for Oncohematology, Department of Medicine, Faculty of Medicine, University of Geneva, Geneva, Switzerland

**Keywords:** sorafenib, maintenance, HSCT = hematopoietic stem cell transplant, acute myeloid leukemia, drug toxicity and adverse effect

## Abstract

Sorafenib significantly improves survival of FLT3-ITD mutated AML patients when used as a post-allogeneic HSCT maintenance. Importantly, clinical trials reported a low rate of toxicities requiring sorafenib discontinuation. The aim of our analysis was to evaluate the real-world experience in patients treated with post-allogeneic HSCT sorafenib maintenance therapy for FLT3-ITD AML with a particular focus on tolerability and toxicity-related treatment interruption. We conducted a single-center retrospective study on 30 FLT3-ITD AML patients undergoing allogeneic HSCT in complete remission between 2017 and 2020 and who received sorafenib maintenance. 26 patients (87%) experienced toxicities leading to dose reduction (n=9) or direct interruption (n=17). Average time on sorafenib was 125 days (range 1-765). Most common toxicities were skin, gastrointestinal, and hematologic. Among patients who had a dose reduction, 4 eventually interrupted the drug and 5 were able to continue. Among patients who interrupted sorafenib because of toxicities, 7 were re-challenged with good tolerance in 3 cases. Overall, 18 patients (60% of the entire cohort) definitively discontinued sorafenib because of toxicities. 14 patients were thereafter switched to midostaurin. Importantly, with a median follow-up of 12 months, the median overall survival was not reached suggesting a positive impact of sorafenib maintenance despite the high rates of treatment interruption. In conclusion, our real-world analysis reveals high rates of toxicity-related interruption of sorafenib maintenance after allogeneic HSCT. Interestingly, our results suggest the feasibility of re-challenging with sorafenib and/or of switching to other maintenance approaches in case of intolerance.

## Introduction

FLT3-Internal Tandem Duplication (FLT3-ITD) mutations of the gene encoding the FLT3 tyrosine kinase receptor are found in 25-30% of AML patients. It is associated with a high risk of relapse and therefore with poor prognosis despite intensive chemotherapy and allogeneic HSCT (1). FLT3 tyrosine kinase inhibitors (TKI) recently emerged as an efficient boost to conventional AML induction chemotherapy, significantly improving survival of FLT3 mutated AML patients in large prospective trials (2). In patients who relapsed after allogeneic HSCT, several studies showed that sorafenib, a broad-spectrum TKI with strong activity against FLT3, induced durable remissions (3). Post-HSCT maintenance with sorafenib emerged in recent years as a way to improve prognosis by diminishing relapse risk of FLT3-ITD AML, as reported in early studies (4–6) as well as phase II (7) and III (8) clinical trials. Overall, retrospective studies and clinical trials reported relatively low rates of drug interruption or reduction suggesting this treatment is well tolerated in the post-transplant setting. Based on these promising outcomes, maintenance with sorafenib is routinely used in many centers for patients with FLT3-ITD AML after allogeneic HSCT, starting as early as hematological reconstitution. The aim of our single-center retrospective analysis was to evaluate the real-world experience in patients treated with post-allogeneic HSCT sorafenib maintenance therapy for FLT3-ITD AML with a particular focus on tolerability and toxicity-related treatment interruptions.

## Methods

### Study design

Our study included 30 patients who received transplantation at our center between 2017 and 2021 for AML with FLT3-ITD in complete hematological remission. Clinical data were retrospectively extracted from the medical records. Written informed consent was obtained from all patients included in the study. All patients included received Sorafenib maintenance therapy starting at time of hematological reconstitution after transplantation. Sorafenib was started at 200 mg BID and increased at 400 mg BID after a week in case of good tolerance. Treatment was planned for two years after transplant, if well tolerated. In case of toxicity, the drug was either reduced to 200 mg BID, or stopped depending on the severity of the toxicity.

### Statistical analysis and data visualization

Baseline characteristics were descriptively reported. Categorical variables were expressed as proportions. Continuous variables were expressed as median with range. Overall survival (OS) was calculated from the date of transplant to death or last follow-up. Progression-free survival (PFS) was calculated from the date of HSCT until disease relapse/progression, death or last follow-up. Probability of OS and PFS were calculated using the Kaplan-Meier estimator. Statistical analyses were performed using R version 3.5.1 with R studio version 1.1.453.

## Results

### High rates of toxicity leading to Sorafenib dose reduction or drug interruption

**Figure 1:**
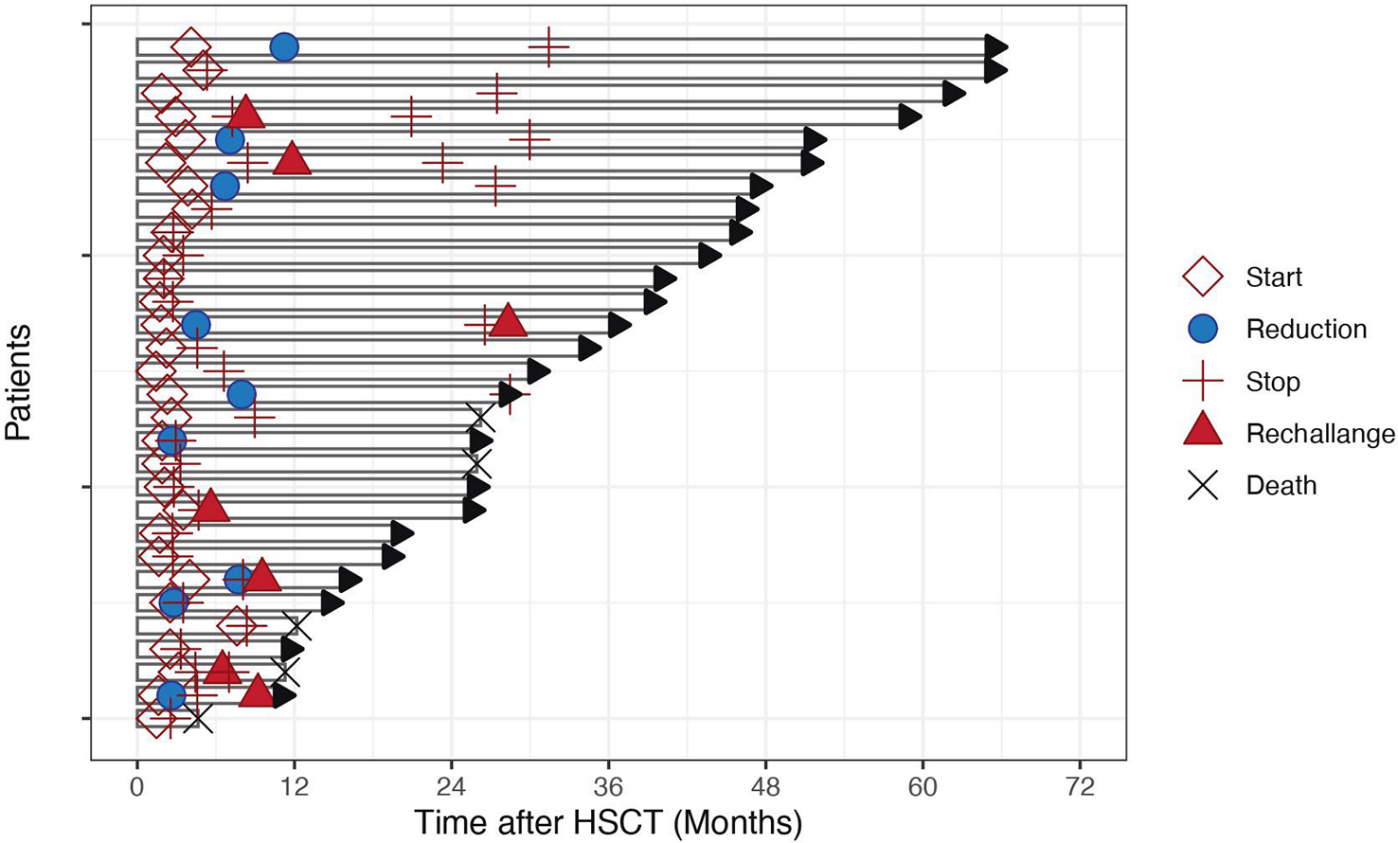
Length of Sorafenib administration in 30 patients over time. Each bar represents the time from transplant date to last follow-up. Symbols on each line indicate sorafenib start, reduction, stop, rechallenge and death dates, if applicable.

Patient characteristics are reported in **Table 1**. Median age at transplant in our cohort was 55 years (29–68). All patients had a FLT3-ITD mutation and 23 (77%) had a NPM1 mutation. Twenty-seven (90%) were transplanted in first complete remission (CR) and 3 (10%) in second CR. At the time of transplant molecular Measurable Residual Disease (MRD) was positive in 13 (43%) and negative in 17 (57% patients. Twenty-one (70%) patients had a comorbidity index of 0 to 2 points and 9 (30%) of 3 points or more. 9 (30%) patients received a graft from an HLA identical donor, 17 (57%) patients received a graft from a matched unrelated donor (MUD), and 4 (13%) from a haplo-identical donor. Sixteen (53%) patients received myeloablative conditioning (MAC) mostly fludarabine (150 mg/m^2^) and treosulfan (42 mg/m^2^). Fourteen (47%) patients received reduced intensity conditioning (RIC) mostly fludarabine (150 mg/m2) and treosulfan (10 g/m^2^). GVHD prophylaxis consisted of Calcineurin Inhibitors (CNI) and Mycophenolate Mofetil (MMF) in 3 (10% patients), CNI and Methotrexate in 19 (64%) patients, CNI, MMF and post-transplant Cyclophosphamide in 6 (20%) patients, CNI, sirolimus and MMF in 2 (6%) patients. Twenty (68%) patients received *in vivo* T-cell depletion before transplantation, with antithymocyte globuline. Eight (27%) patients received ex-vivo T-cell depletion with anti-CD52 antibody in the bag. Median follow-up was 324 (62–1099) days.Median time from transplant to sorafenib initiation was 63 (41–213) days. At sorafenib start, median hemoglobin was 112 g/dl (77–152), median platelet count was 170 G/l (49–278), median leucocyte count was 4.8 G/l (1.55-14.3), median renal clearance measured with GFR was 80 ml/min/m^2^ (49–117). Twenty-six (87%) patients experienced toxicities leading to drug interruption in 17 patients and dose reduction in 9 patients (**Figure 1**).

**Table 1:** Patient characteristics.

Median age (range), years	55 (29–68)
Sex, *n* (%)	
Male	15 (50)
Female	15 (50)
Mutational status, *n* (%)	
FLT3-ITD mutation	30 (100)
NPM1	23 (77)
Status at transplant	
1^st^ CR, *n* (%)	27 (90)
2^nd^ CR, *n* (%)	3 (10)
Molecular MRD status at transplant	
MRD positive	13 (43)
MRD negative	17 (57)
HCT-CI, *n* (%)	
0-2pts	21 (70)
≥ 3pts	9 (30)
Conditioning Type, *n* (%)	
MAC	16 (53)
RIC	14 (47)
GVHD prophylaxis regimen	
CNI, NMF	3 (10)
CNI, MTX	19 (64)
CNI, MMF, PTCy	6 (20)
CNI, sirolimus, MMF	2 (6)
T-cell depletion	
ATG	20 (68)
Ex-vivo T-cell depletion	8 (27)
No T-cell depletion	7 (24)
Donor type, *n* (%)	
Sibling donor	9 (13)
Matched-unrelated	17 (57)
Haplo-identical	4 (13)
Stem cell source	
Peripheral blood	29 (97)
Bone marrow	1 (3)
Median time to sorafenib initiation(range), days	63 (41-213)
Laboratory values at sorafenib start	
Median WBC (range), G/I	4.8 (1.5-14.3)
Median Platelets (range), G/I	170 (49-278)
Median hemoglobin (range), g/dl	112 (77-152)
Median renal eGFR (range), ml/min/m^2^73	80 (49-117)

FLT3, fms-like tyrosine kinase 3; NPM1, nucleophosmin 1; CR, complete remission; MRD, Measurable Residual Disease; HCT-CI, hematopoietic cell transplant comorbidity index; MAC, myeloablative conditioning; RIC, reduced intensity conditioning; CNI, calcineurin inhibitors; MMF, mycophenolate mofetil; MTX, methotrexate, ATG, Antithymocyte globulin; HLA, human leucocyte antigen; WBC, white blood cell; eGFR, estimated glomerular filtation rate; PTCy, post-transplant cyclophosphamide.

### Heterogeneous profile of toxicities requiring sorafenib dose reduction or interruption

**Figure 2:**
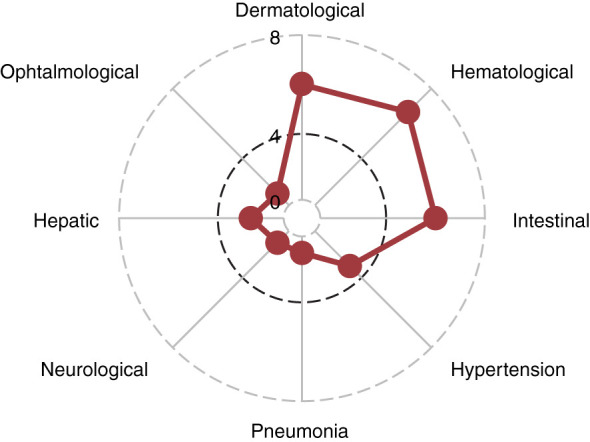
Spider Plot of sorafenib toxicities. Each point on the disc indicates the number of patients in the cohort who experienced each type of toxicity.

In the 26 patients with reported toxicities, most common toxicities were skin (n=5, grade II), gastrointestinal (n=7, 27%, grade II and III), and hematological (n=7, 27%, grade III). One patient experienced concomitant uveitis (grade III) and pneumonia (grade IV), both resolved after sorafenib interruption. 3 (11%) patients experienced hypertension (grade II in 2 patients and III in 1 patient), 2 (7%) had hepatitis (1 grade II and 1 grade III) and one (4%) patient had a PRESS (posterior reversible encephalopathy syndrome) grade IV possibly related to sorafenib (**Figure 2**). Skin biopsies were obtained in three patients who presented with an erythematous and papular rash with follicular hyperkeratosis. In the three of them, a lymphocytic infiltrate was present surrounding the hair follicules, with presence of polynuclear granular leucocytes. In one patient eosinophils infiltration and keratinocyte necrosis were present. For this reason, acute skin GvHD could not be excluded in this patient but the condition rapidly improved after sorafenib interruption. Of note, we observed no hand-foot syndrome nor stomatitis, which are the most common cutaneous side-effects reported with sorafenib (9). Hematological adverse events were grade II and III thrombocytopenia in 6 patients and grade III neutropenia in 1 patient. Gastro-intestinal symptoms included diarrhea in 2 patients, abdominal discomfort in 1, dysgeusia in 1 patients, nausea in the other patients. No digestive biopsies were performed in any of the patients because symptoms were rapidly resolved after sorafenib interruption or dose adjustment.

### Patient care and outcome after sorafenib interruption or reduction

Of the 9 patients (30% of entire cohort) who had a dose reduction, 4 eventually stopped because of toxicity and 5 continued the drug. Median time on sorafenib before interruption in the whole cohort was 41 days (range 1-765).

Among 21 patients (70% of entire cohort) who interrupted (either directly for 17 patients or after reduction attempt for 4 patients) sorafenib because of toxicities, 7 were re-challenged with good tolerance in 3 cases and 4 eventually stopped because of toxicity recurrence.

In the end, definitive discontinuation because of toxicities happened in 18 patients (60% of entire cohort). Non-toxicity-related causes of sorafenib discontinuation were relapse in 3 patients, including FLT3-ITD negative relapse in 1 patient, and end of scheduled maintenance in 5 patients. Among patients who discontinued the drug because of toxicities, 14 patients were switched to midostaurin. Among them, 5 are still taking the drug, 3 completed the 2-year maintenance and 6 interrupted midostaurin because of toxicities.

**Figure 3:**
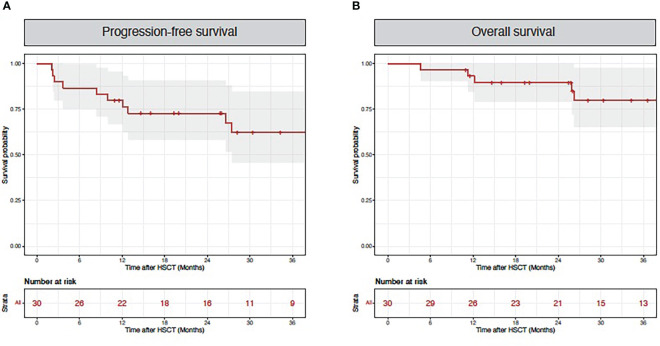
Patient outcomes. Red lines indicate progression-free survival **(A)** and overall survival **(B)** for the entire cohort.

Importantly, the analysis of patients’ outcome showed a favorable progression-free survival (24-month PFS 73% (58%-91%); **Figure 3A**) and overall survival (24-month OS was 90% (79%-100%); **Figure 3B**) despite the high rate of treatment interruption.

## Discussion

Relapse remains the first cause of death after allogeneic HSCT in AML patients. The rationale of maintenance therapy is to reduce the risk of relapse without impeding the graft-versus-leukemia (GvL) effect. Based on impressive survival benefits demonstrated in phase II and III studies, the use of sorafenib maintenance has rapidly expanded in recent years. Sorafenib is a first-generation multi-target FLT3 tyrosine kinase inhibitor that has been used since 2007 in oncology, for treatment of advanced hepatocellular carcinoma and renal carcinoma. Besides FLT3, it mainly targets vascular endothelial growth factors (VEGFR1-3) and Raf kinases, but additional reported targets include KRAS or BRAF (10–12). Most common treatment related toxicities include hypertension, diarrhea, fatigue, hand-food skin reaction (13). In trials for advanced solid cancers, these side-effects were mostly mild, with less than 10% of high-grade side effects. In one phase III trials that included 903 patients with renal carcinoma (14) and two phase III trials that included 602 and 226 hepatocarcinoma patients treatment discontinuation rates ranged from 10 to 38% (15, 16).

Early retrospective studies on sorafenib maintenance as well as the phase I clinical trial reported a fairly good tolerability, with dose reduction rates ranging from 15 to 27% and interruption rates ranging from 18-31% (4–6). In a retrospective series, Chappell et al. reported a higher rate of dose reduction (66%) with a low (14%) rate of drug interruption (17).

In the two randomized trials, dose reduction rates happened in nearly half of patients but drug interruption and discontinuation rates were much less frequent than in our experience. In the phase II randomized, placebo-controlled phase II SORMAIN trial dose reduction was performed in 48.8% of patients and discontinuation in 22% of patients in sorafenib group (7). In the phase III trial conducted by Xuan et al, dose reduction rate was 42% in sorafenib group while dose interruption rate was 12% and definitive discontinuation rate was only 5% (8).

In our real-world analysis of sorafenib maintenance after allogeneic HSCT, dose modifications, especially interruption, because of toxicities, were particularly high: reduction rate was 30% of entire cohort, interruption rate (direct or after reduction attempt) was 70% of entire cohort and definitive discontinuation rate was 60% of entire cohort. Such high rates of toxicity-related dose adjustments or interruptions are closer to the ones reported by Pratz et al. in their single-arm pilot study where sorafenib dosing was individualized, starting at a dose of 200 mg/day and titrated based on tolerability and toxicities (18). In this study, which included 44 patients (median age 52 years, very close to our own cohort) treated with sorafenib post-transplant, most patients (40/44, 90%) were unable to escalate the dose to reach 400 mg BID, with only 4 patients able to tolerate 400 mg BID. The authors also performed elegant pharmacokinetics and pharmacodynamics studies where they measured sorafenib concentrations at different timepoints (accounting for sorafenib active metabolite) and assessed FLT-ITD inhibition with a plasma inhibitory activity (PIA) assay. Interestingly, these correlative studies found consistent inhibition of FLT3 at all tolerability-determined dosing levels. Based on these results, the authors recommend an individualized dosing for patients after transplantation, according to tolerability. In our cohort, sorafenib was started at 200 mg bid and, after a week, increased at 400 mg. After this rather quick dose increase, in case of suspected toxicities, the drug was more frequently interrupted than decremented first. A first explanation for this is a low tolerance to side-effects in this heavily pre-treated population of transplanted patients. In addition, the high rate of drug interruption we found in the real-world setting may be due to the fact that two of the most frequent side effects of sorafenib we observed were gastro-intestinal and cutaneous, both of which are very frequent sites of acute GVHD. One common strategy when suspecting this complication is to stop any medications that could be causing the symptom and, if persistent, proceed to biopsies to document GVHD.

Only 7/21 patients interrupting sorafenib in our series were rechallenged thereafter, while the 14 remaining patients were not rechallenged because of fear of recurrence of side-effects and relatively easy access to midostaurin as an alternative FLT3 inhibitor. Although among the 14 patients who switched to midostaurin the majority of them experienced adverse events when exposed to this alternative multitargeted TKI, nearly half were able to continue the drug. Although this drug has been proven to be effective in first-line therapy, data supporting its use as a post-allogeneic HSCT maintenance are still limited. In the RADIUS trial, a phase II randomized study to investigate the role of midostaurin maintenance, an early report found a benefit to adding midostaurin to SOC as a maintenance treatment in patients with FLT3-ITD AML after allogenic HSCT (19). The estimated 24-month RFS was 85% (64-94%) and the estimated 24-month OS was 85% (65-94%) in the midostaurin arm. Dose adjustments occurred in 63% of patients (related to adverse events in 84% cases) and treatment discontinuation occurred in 27% of patients, mostly due to gastro-intestinal adverse events (nausea and vomiting) or liver enzyme elevation. Gilteritinib is a newer generation TKI with specific and potent activity against FLT3-ITD and AXL1-kinases (20), currently under investigation as a maintenance treatment in the BMT CTN Protocol 1506, and results are awaited regarding efficacy and tolerance in the post-transplant maintenance setting to see if it can replace sorafenib in this indication. Importantly, the analysis of patients’ outcome in our cohort confirmed the previously reported positive impact of sorafenib maintenance on overall survival despite high rates of treatment interruption: 24-month PFS was 73% (58%-91%) and 24 months OS was 90% (79%-100%). These outcomes are comparable to what was found in the randomized trials: in the SORMAIN trial 24-months RFS was 85% and 24-month OS was 90.5% in the sorafenib group (7). In phase III trial by Xuan et al, the authors found comparable outcomes 24-months RFS of 78.9% and 24-months OS of 82.% (8). We can hypothesize that sorafenib impact, despite the relatively short treatment course in most patients, could be due to a long-lasting immune-mediated effect. In a preclinical study, sorafenib was shown to promote a graft-versus-leukemia effect by inducing the secretion of T and NK cells growth factors, namely IL-15 by AML cells (21). Subgroup analysis done in the phase III trial revealed that patients who received allo-HCT from matched sibling donor and in patients without GVHD, retained the strongest benefit from sorafenib, also suggesting an immunomodulatory role (8). A non-mutually exclusive hypothesis is that the positive outcome observed can be due to alternative maintenance therapies in patients who were unable to tolerate sorafenib.

In conclusion, our real-world experience with sorafenib maintenance therapy after allogeneic HSCT reveals higher rates of toxicity-related dose reduction and drug interruption than previously reported in clinical trials. Importantly, we confirmed the benefit of the drug, despite high-interruption rates potentially as a consequence of the immunomodulatory role of sorafenib and/or of the feasibility of switching to midostaurin in case of intolerance.

## Data availability statement

The raw data supporting the conclusions of this article will be made available by the authors, without undue reservation.

## Ethics statement

The studies involving human participants were reviewed and approved by Commission Cantonale d’Ethique de la Recherche sur l’être humain de Genève. The patients/participants provided their written informed consent to participate in this study. Written informed consent was obtained from the individual(s) for the publication of any potentially identifiable images or data included in this article.

## Author contributions

SM, FS, and YC designed the study. SM, FG, A-CM, AP, and SM-L collected the clinical data. SM and FS analyzed the data, performed statistical analysis, and prepared figures. SM wrote the manuscript. FS and YC provided overall guidance and edited the manuscript. All authors contributed to the article and approved the submitted version.
